# Grazing Altered the Pattern of Woody Plants and Shrub Encroachment in a Temperate Savanna Ecosystem

**DOI:** 10.3390/ijerph16030330

**Published:** 2019-01-24

**Authors:** Zhiyong Zhang, Bo Zhang, Xiao Zhang, Xiaohui Yang, Zhongjie Shi, Yanshu Liu

**Affiliations:** 1 Institute of Desertification Studies, Chinese Academy of Forestry, Beijing 100091, China; zzy100083@163.com (Z.Z.); zhangxiao978@caf.ac.cn (X.Z.); yangxh@caf.ac.cn (X.Y.); 2 Research Institute of Forestry Policy and Information, Chinese Academy of Forestry, Beijing 100091, China; 3 Beijing Station of the Forestry Science and Technology Extension, Beijing 100029, China; zbbeilin@126.com

**Keywords:** *Ulmus pumila*-dominated temperate savanna, population structures, spatial patterns, spatial association, grazing pressure, shrub encroachment

## Abstract

*Ulmus pumila*-dominated temperate savanna is an important tree-grass complex ecosystem in the Otindag sand land, northern China. To date, few investigations have been undertaken on the spatial patterns and structure of this ecosystem and its driving factors under different grazing pressures. The objective of our study therefore is to explore whether grazing has affected the population structure/pattern of woody plants and shrub encroachment in a temperate savanna ecosystem. Results indicate that species richness and seedlings decreased with increasing grazing pressure. An increase in grazing pressure did not significantly affect adult-tree density, but it hindered the normal regeneration of *U. pumila* seedlings, further inducing population decline. *U. pumila* seedlings had a more significant aggregated distribution than juvenile or adult trees. The adult and juvenile trees had an aggregated distribution at the small scale and a random distribution at the large scale. Shrubs also showed a significant aggregated distribution. No clear effect on the spatial patterns of adult trees was observed; however, there was a noticeable effect for juveniles and seedlings under different grazing pressures. *U. pumila* seedlings had a positive association with their juveniles and *Spiraea aquilegifolia,* but a negative association with *Caragana microphylla*. Shrub encroachment occurred with decreasing grazing pressures. In conclusion, overgrazing led to the decline of *U. pumila* population, but the decrease in grazing pressure increased shrub encroachment in the temperate savanna ecosystem. Moderate grazing management may be a better way to enhance the stability of *U. pumila* population and reduce shrub encroachment.

## 1. Introduction

Savanna patchy ecosystems are predominantly structured by complex interactions between the dominant trees (or shrubs) and their herbaceous plants found beneath or around trees [1,2,3]. Savanna ecosystems, which are found in temperate and tropical regions in Africa, Australia, Asia, and America, account for about 12% of the world’s total area [4]. In China, temperate savanna ecosystems are mainly distributed in the ecotones of arid and semi-arid areas [5]. As a typical temperate savanna ecosystem, *Ulmus pumila*-dominated savanna is a climate climax community with a characteristic tree-grass two-phase structure that is composed of *U. pumila*-dominated woody plants and herbaceous understory vegetation. These ecosystems are widely distributed in the Otindag and Horqin sand lands of northern China [6,7], and they are ecologically important for sand stabilization and the provision of small habitats for livestock [7].

Historically, the *U. pumila*-savanna ecosystem of the Otindag sand land was an important pasture source, providing a large amount of forage for livestock. In recent years, serious desertification has occurred in this area due to a range of changing conditions, such as climate change, land use change, and economic development [8,9,10]. Although precipitation and soil moisture have been the main limiting factors affecting population regeneration of woody plants in arid and semi-arid areas [11], grazing has become an important factor affecting population regeneration; long-term continuous heavy grazing in savanna ecosystems has resulted in the destruction of topsoil and degradation of the rangeland [12,13]. These changes have also affected the regeneration and survival of woody plants [14,15]. In the Otindag sand land, due to severe damage to the *U. pumila*-dominated temperate savanna ecosystem, especially for woody vegetation, ecological degradation has occurred, such as decreasing temperate savanna area, loss of structural integrity, poor population regeneration, and changing spatial patterns [16,17,18]. Scientific focus on the degradation of this temperate savanna, especially the effects of overgrazing on *U. pumila* population regeneration and structure, has been widely overlooked [15,19].

Structural and spatial patterns are the most important characteristics of a plant population [20,21]. Population structure, defined as the distribution of individuals of different sizes and ages within a population, can reflect the age composition, generational characteristics, and future changes at a given time [22,23]. Spatial pattern refers to the position and distribution of individual plants in a population or ecosystem, reflecting the population distribution and intra- or inter-specific associations, and it can be used to investigate potential ecological processes [24,25]. In recent years, the analysis of population structure and spatial patterns has become an important tool to understand population regeneration, community stability, and the interactions between plants and their environment across the dimensions of time and space [26,27,28,29].

Controlled-grazing management is one of the most important ecological management methods in rangeland ecosystems [30,31]. To restore degraded rangeland, the Chinese government has undertaken an ecological restoration program over the last 15 years that has included the conversion of farmland to rangeland in the desertification-prone area of northern China [32]. In this study, our objectives are to explore the effects of grazing pressures on *U. pumila* population structure, spatial patterns, and interactions among the woody plants in the Otindag sand land using field observation and point pattern analysis. The specific aims of our investigation are to examine if: (1) increasing grazing pressure hinders the regeneration of woody plants and further affects population structure; (2) the strong interference of grazing affects the pattern and interactions of woody plants; and, (3) overgrazing is the main cause of shrub encroachment in a temperate savanna ecosystem. This investigation will provide a better understanding of the degradation processes affecting *U. pumila*-dominated temperate savanna, as well as a theoretical basis for degraded rangeland restoration and management of livestock and grazing lands.

## 2. Study Area and Methods

### 2.1. Study Area

**Figure 1:**
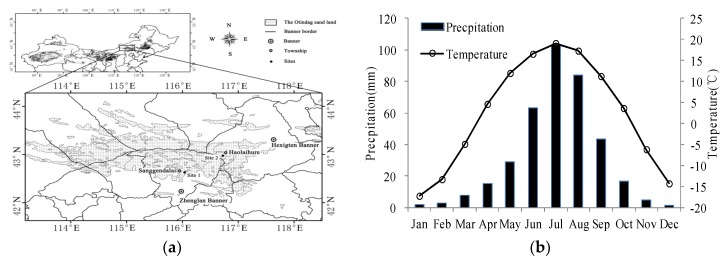
The location (**a**) and climate (**b**) of the study area.

This study was conducted at the Sanggendalai Township (42°39′13.54″ N, 116°00′12.68″ E) of Xilin Gol League, and the Haolaihure Township (42°54′47.68″ N, 116°42′22.27″ E) of Chifeng City, located in the central and eastern Otindag sand land, Inner Mongolia, respectively (Figure 1). The Otindag sand land is about 340 km long (from east to west) and 30–100 km wide (from 42°10′ N and 112°10′ E to 43°50′ N and 116°30′ E) [33]. The area has a typical temperate continental semi-arid climate with warm summers and cold winters. The average annual temperature is about 1.8 °C and annual precipitation ranges from 250 to 350 mm, 80% of which occurs between June and August [19]. The soil type is aeolian sandy soil with a mean depth of 200 cm and a calcic horizon occurring at a depth of 30–100 cm. The water table has been deepened to 1.0–1.5 m below ground level [34]. The two sites in this study have similar climate, geography, and soil conditions.

In this area, *U. pumila* is dominant, with other woody species including *Caragana microphylla* Lam., *Spiraea aquilegifolia* Pall., *Ribes diacanthum* and *Salix linearitipularis,* and herbaceous plants, including *Leymus chinense* (Trin.) Tzvel., *Cleistogenes squarrosa* (Trin.) Keng (Gramineae), *Agropyron cristatum* (Linn.) Gaertn., *Polygonum divaricatum* L., *Artemisia frigida* Willd. Sp. PI., *Leymus secalinus* (Georgi) Tzvel., *Potentilla chinensis* Ser., and *Allium mongolicum* Regel (Liliaceae) [35]. The main land-use in the area is grazing or mowing and the livestock are cattle and sheep. To prevent rangeland degradation and land desertification, different grazing methods have been employed in this area, including annual enclosure, seasonal grazing, annual grazing, etc.

### 2.2. Data Collection

To investigate the effects of different grazing methods on the spatial distribution of *U. pumila*, sample plots were selected in a flat area with a relatively low topographic relief (<1 m), thus eliminating the effect of habitat heterogeneity that is caused by the undulating dunes. To examine the effect of different grazing pressures on the *U. pumila*-savanna ecosystem, three rangelands were selected: (i) lightly-grazed rangeland: for 18 years, this area was grazed in the winter and not disturbed in other seasons; (ii) moderately-grazed rangeland: for 20 years, this area was fenced in the summer to exclude livestock and seasonal grazing occurred in the winter and spring, while it was mowed in the autumn; and, (iii) heavily-grazed rangeland: for 25 years, this area was public rangeland that was accessed all year for grazing.

**Figure 2:**
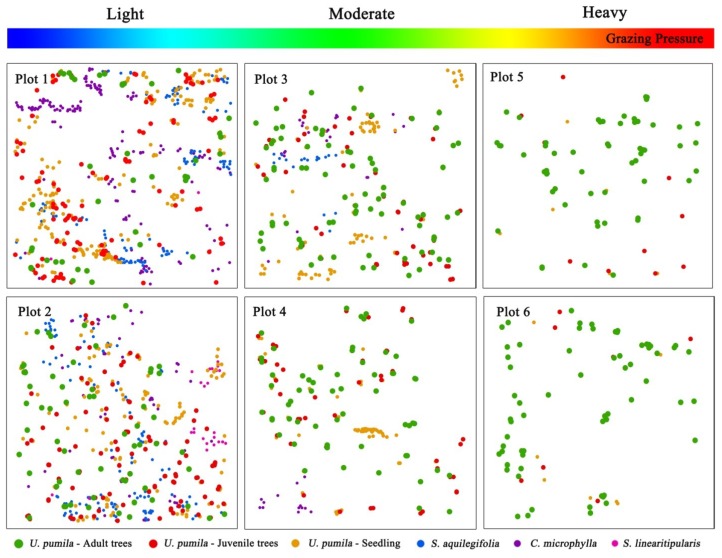
Spatial distribution of the main woody plants in the different grazing plots.

Six sample plots with a size of 100 × 100 m (numbered as Plots 1–6) were established in the different grazing pressure areas in September, 2016 (Figure 1). Plots 1, 3, and 5 were located in the Sanggendalai Township and Plots 2, 4, and 6 were located in the Haolaihure Township. Plots 1 and 2 were located in the lightly-grazed rangeland; Plots 3 and 4 in the moderately-grazed rangeland; and, Plots 5 and 6 were in the heavily-grazed rangeland (Figure 2). To ensure consistency in the study, each site had similar conditions (such as soil type, geomorphology, and precipitation). Each sample plot was further divided into 25 subplots (20 × 20 m), and all woody plants in each subplot were measured and identified; trees with a diameter at breast height (DBH) ≥1 cm were marked. The species names, DBH, total height, clear-bole height, and crown diameter of all woody plants (trees and shrubs) were recorded. All elm seedlings (DBH < 1 cm) in each plot were recorded, including the heights and coordinates. The coordinates of woody plants in each plot were recorded using an Electronic Total Station with the southwestern corner of the plot as the origin. Additionally, in the center of each subplot, a small plot with a size of 1 m × 1 m was selected where all herbaceous vegetation was identified; the area of cover and number of species were also recorded. After recording this information, all grasses in aboveground were harvested to determine their biomass. The spatial distribution of woody plants is shown in Figure 2 and Table 1 details the plot characteristics.

### 2.3. Data Analysis

#### 2.3.1. Population Structure

In a specific environment, the response of the age class of woody plants to its environment is consistent with the response of the DBH to it environment [36], meaning that the DBH of woody plants can reflect the age class of the population. Therefore, the diameter classes in our study were used to explain the age structures of the main tree species on the basis of the age dependence of tree diameter. According to the correlation between the diameter and age of *U. pumila* in the Otindag sand land [37], all of the trees were divided into 10 categories according to their DBH: Class I (without DBH), Class II (DBH < 5cm), Class III (5 cm ≤ DBH < 10 cm), Class IV (10 cm ≤ DBH < 15 cm), Class V (15 cm ≤ DBH < 20 cm), Class VI (20 cm ≤ DBH < 25 cm), Class VII (25 cm ≤ DBH < 30 cm), Class VIII (30 cm ≤ DBH < 35 cm), Class IX (35 cm ≤ DBH < 40 cm), and Class X (DBH ≥ 40 cm). The number and percentage of *U. pumila* individuals in each DBH class were calculated and mapped in each plot. Gaussian and polynomial models were used for data testing and curve fitting to infer population structure in the future.

To analyze the effect of grazing on herbaceous plants, the number of herbaceous species was counted from 25 samples in each plot. The average biomass and standard error in each plot was calculated from 25 observed herbaceous samples.

#### 2.3.2. Point Pattern Analysis

Spatial pattern analysis categorized *U. pumila* into three classes by size: seedlings (without DBH), juvenile trees (DBH < 20 cm), and adult trees (DBH ≥ 20cm). If the number of individuals in a category was very small, this category was excluded from spatial pattern analysis.

Point pattern analysis, as proposed by Ripley [38] and improved by Diggle [39], is an effective tool in ecological studies. Ripley’s *K*-function, expressed as the expected number of points in a circle of radius *r* centered at an arbitrary point divided by the intensity *λ* of the pattern, has been widely used in plant analysis to characterize the spatial patterns of species [40,41]. However, as the function is a cumulative measure incorporating information from both large and small scales, the function can confound large-scale effects with small-scale effects [40,42,43].

The pair correlation function *g*(*r*), which arises if the circles of Ripley’s *K*-function are replaced by rings, provides the expected number of points at distance *r* from an arbitrary point, divided by the intensity *λ* of the pattern. The function *g*(*r*) is a probability density function for the interpretation of neighborhood density and it is sensitive to small-scale effects that can eliminate the cumulative effect that is caused by the *K*(*r*) function. Similar to Ripley’s *K* function, the function *g*(*r*) includes both univariate and bivariate statistics. The univariate statistic is used to analyze the spatial pattern of one object, while the bivariate statistic is used to analyze the spatial association of two objects (pattern 1 and pattern 2) [40]. In this study, the univariate *g*(*r*) was used to analyze the spatial pattern of woody plants and the bivariate *g*_12_(*r*) was used to analyze the spatial associations between the *U. pumila* trees and shrubs, as well as between the different growth stages (i.e., seedling, juvenile, and adult stage) of *U. pumila* trees.

In our study, the impacts of habitat heterogeneity on point pattern analysis, including soil and topography, were eliminated by locating all plots on flat areas. Therefore, we used the null model of complete spatial randomness (CSR) as a null hypothesis to check the level of significance. In the univariate analysis, if *g*(*r*) higher than the upper confidence limit indicates aggregation, *g*(*r*) lower than the lower confidence limit indicates regularity [40]. In the bivariate analyses, when considering the fact that large trees may impact the distribution pattern of small trees within their area of influence (competition), we used a bivariate g function for large trees and small trees or trees and shrubs using both toroidal shift and the antecedent condition null model options [40]. This tests whether one pattern (small trees) is influenced by a second pattern (large trees) and it assesses whether there are more (or fewer) small trees in the neighborhood of large trees than expected under a random distribution of small trees [44]. In analyzing the interaction between trees and shrubs, we examined the spatial association between the two species with an independent null model [40]. If values of *g*_12_(*r*) are above the upper (or below the lower) limit of the confidence envelope, this indicates that pattern 2 is positively (or negatively) associated with pattern 1 at a given distance *r*. If values of *g*_12_(*r*) are within the estimated upper and lower envelopes, this indicates that there is no interaction between patterns 1 and 2.

Point pattern analysis was performed using Programita 2014 software (Leipzig, Germany) [40]. To eliminate the influence of edge effects on the point pattern analysis, spatial scale was assigned as the half length of plots (0–50 m) with a step of 1 m. The 99% confidence envelopes for each null model were calculated by running 199 Monte–Carlo simulations. Figures were plotted using ArcGIS 10.2 (Redlands, California, USA) and Origin 8.0 (Northampton, MA, USA).

## 3. Results

### 3.1. Stand Structure

When grazing pressures increased from lightly-grazed to heavily-grazed conditions, herbaceous species respectively decreased from 20 and 21 species (Plots 1 and 2) to 14 and 15 species (Plot 5 and 6), and aboveground herbaceous biomass declined from 127.63 and 225.85 g·m^−2^ (Plots 1 and 2) to 80.84 and 103.02 g·m^−2^ (Plots 5 and 6) (Table 1). Three shrub species with a large number of individuals (*C. microphylla*, *S. aquilegifolia*, and *S. linearitipularis*) were observed in the lightly-grazed plots (Plots 1 and 2), while only *C. microphylla* and *S. aquilegifolia* were observed in the moderately-grazed plots (Plots 3 and 4). These shrub species were not observed in the heavily-grazed plots (Plots 5 and 6).

For *U. pumila*, the number of seedlings significantly decreased from 106 and 101 seedlings/ha (Plots 1 and 2, respectively) to six and nine seedlings/ha (Plots 5 and 6, respectively) from the lightly-grazed plots to the heavily-grazed plots. In the moderately-grazed plots, *U. pumila* seedlings accounted for 84.0% and 66.4% (Plots 3 and 4, respectively) as compared to the lightly-grazed plots. Tree densities also declined from the lightly-grazed plots (214 and 187 trees/ha, Plots 1 and 2, respectively) to the heavily-grazed plots (100 and 95 trees/ha, Plots 5 and 6, respectively). However, the average DBH was highest in the heavily-grazed plots and lowest in the lightly-grazed plots, which may be due to fewer small trees being present in the heavily-grazed plots. For adult *U. pumila* trees, there was not a significant change in tree density between the different plots.

**Figure 3:**
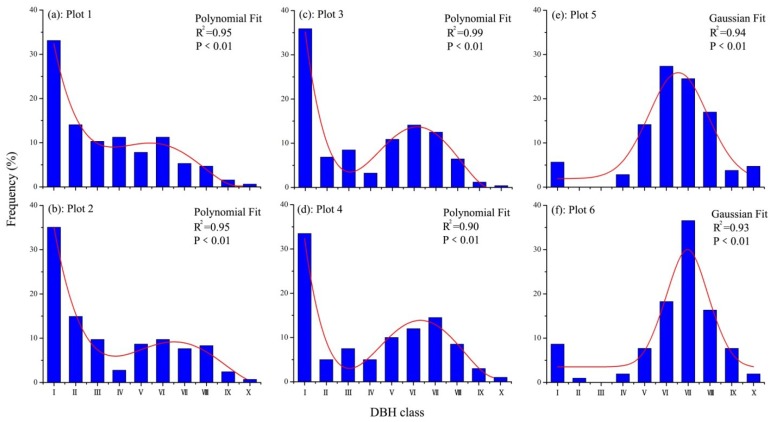
DHB class structure of *U. pumila* trees under different grazing pressures.

According to the polynomial fitting result (Figure 3a–d, polynomial order = 4), *U. pumila* trees had a bimodal DBH distribution under lightly- and moderately-grazed pressures (Plots 1–4). Seedlings (without DBH) and adult trees (DBH ≥ 20 cm) were more abundant in the moderately-grazed plots (Plots 3 and 4) than juvenile trees (DBH < 20 cm), with a mixed unimodal distribution and reversed J shape. In the lightly-grazed plots, *U. pumila* were mainly distributed at smaller DBH classes. DBH had a reverse J distribution, suggesting compatibility with a trend of expanding population size. According to the Gaussian fitting result (Figure 3e,f, Gaussian number peak = 1), the DBH distribution of *U. pumila* in heavily-grazed plots had a unimodal distribution; *U. pumila* trees thrived in the large diameter Class VI (20 cm ≤ DBH < 25 cm) at Plot 5 and in Class VII (25 cm ≤ DBH < 30 cm) at Plot 6. However, Classes II, III, and IV in Plots 5 and 6 were not present. This result suggests a declining *U. pumila* population under heavy-grazing pressures. Therefore, the effects of grazing on the population structures of *U. pumila* were mainly reflected in the damage of juvenile trees and seedlings, with grazing limiting the normal regeneration of the *U. pumila* population and accelerating the process of their decline.

### 3.2. Spatial Pattern and Association of U. Pumila

**Figure 4:**
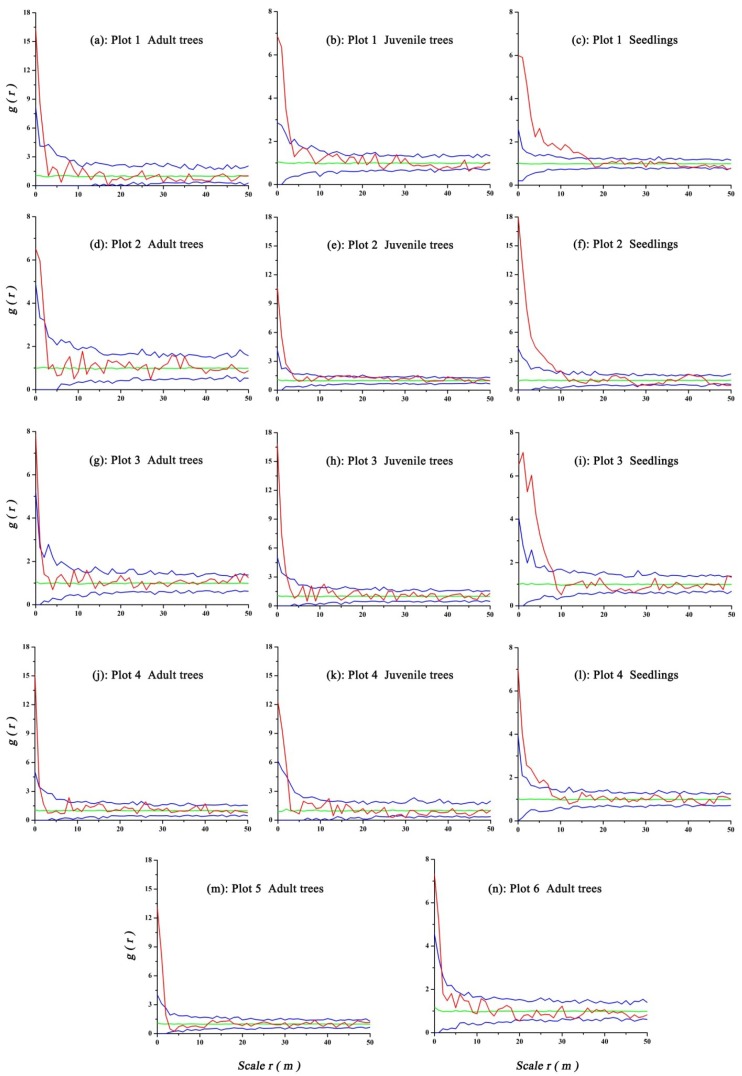
Spatial pattern of the different categories of *U. pumila* using the complete spatial randomness (CSR) null model. The red line indicates the value of the summary function for the data point pattern, the blue line indicates the confidence interval using the Monte Carlo test, and the green line indicates the reference line. The number of simulated point pattern was 99%.

Analysis of the univariate spatial pattern of *U. pumila* using the CSR null model while accounting for antecedent conditions showed that all life stages exhibited small scale aggregations. As spatial scale increased the level of aggregation decreased, and it tended to have a random distribution (Figure 4). The scales of aggregation were different at different life stages. At the 0–2 m scale, adult trees had significant aggregation in Plots 1, 2, 5, and 6, while at the 0–1 m scale, significant aggregation was recorded in Plots 3 and 4. Significant aggregation was recorded for juvenile trees at the 0–3 m scale in Plots 1 and 2, and at the 0–2 m scale in Plots 3 and 4. Plot 1 had significant aggregation at the 0–17 m scale for seedlings and significant seedling aggregation was also observed at the 0–10 m scale in Plot 2 and the 0–8 m scale in Plots 3 and 4. All life stages tended to have a random spatial distribution at all other scales in all of the plots.

**Figure 5:**
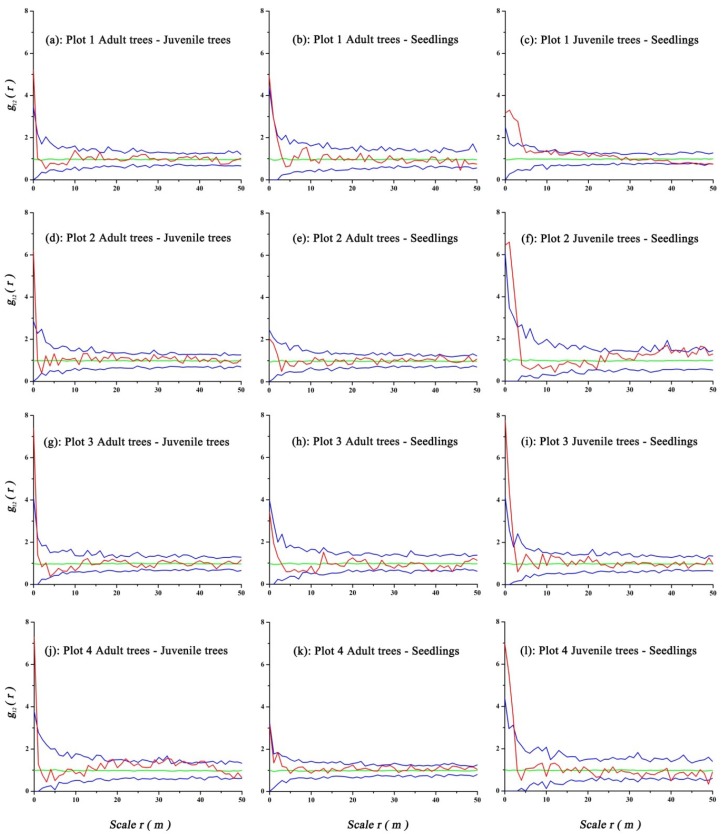
Spatial associations of different life stages of *U. pumila* with the CSR null model. Graph colors are defined in Figure 4.

Bivariate spatial analysis of the different stages of *U. pumila* using the CSR null model and accounting for antecedent conditions found that adult trees had significant positive associations with juvenile trees at the 0-1 m scale, and associations were not significant at other scales in Plots 1–4. Adult trees had significant positive associations with seedlings at the 0–1 m scale in Plot 1, but no associations at all scales in Plots 2–4. Significant positive associations between juvenile trees and seedlings were observed at scales 0–4 m in Plot 1, 0–3 m in Plot 2, and 0–2 m in Plots 3 and 4, and no significant associations were identified at other scales in the four plots (Figure 5).

### 3.3. Spatial Pattern of Shrubs

**Figure 6:**
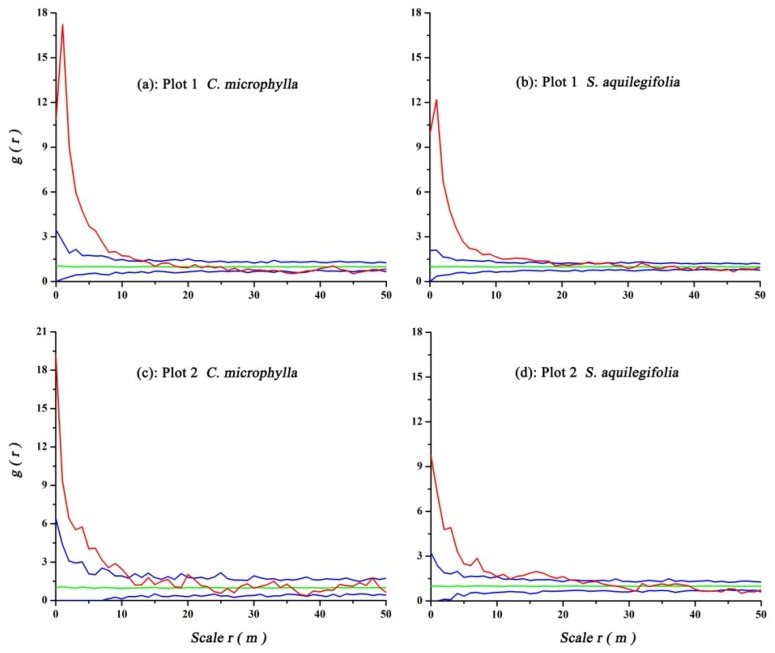
Spatial pattern of different shrubs with the CSR null model. Graph colors are defined in Figure 4.

Univariate spatial analysis of shrubs found that *C. microphylla* had significant aggregation at the 0–14 m scale in Plot 1 and at the 0–12 m scale in Plot 2. *S. aquilegifolia* had significant aggregation at the 0–18 m scale in Plot 1 and at the 0–22 m scale in Plot 2. These two shrubs tended to have a random spatial distribution at all other scales in the two plots (Figure 6).

### 3.4. Spatial Association of Ulmus Pumila and Shrubs

**Figure 7:**
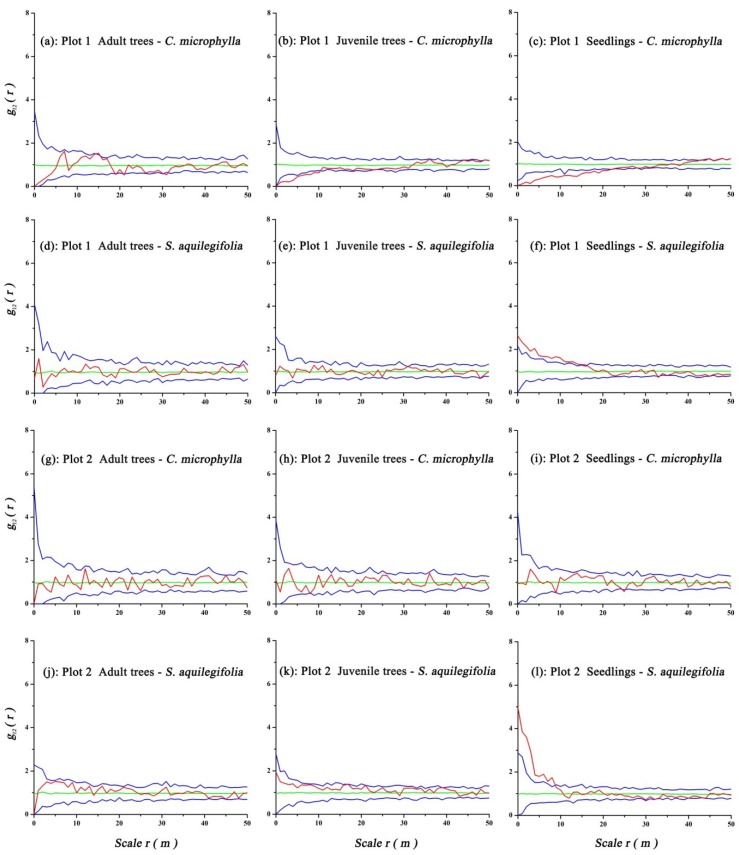
Spatial associations of *U. pumila* trees and shrubs with the CSR null model. Graph colors are defined in Figure 4.

Bivariate spatial analysis of *U. pumila* trees and shrubs using the CSR null model while accounting for antecedent conditions found that adult trees had no significant associations with *C. microphylla* and *S. aquilegifolia* at all scales in Plots 1 or 2. In Plot 1, juvenile trees had significant negative associations with *C. microphylla* at the 0–10 m scale and no significant associations with *S. aquilegifolia* at all scales. Seedlings had significant negative associations with *C. microphylla* at the 0–22 m scale and a positive association with *S. aquilegifolia* at the 0–17 m scale. In Plot 2, juvenile trees had no significant associations with *C. microphylla* and *S. aquilegifolia* at all scales, seedlings had no significant association with *C. microphylla* at all scales, and *S. aquilegifolia* had a significant positive association with seedlings at the 0–9 m scale (Figure 7).

## 4. Discussion

### 4.1. Effects of Grazing on Stand Structure

Takahashi et al. [45] noted that the population structure of a species reflects its regeneration processes. When compared to the spatial structure of a stand, insights into the population dynamics and future trends of a stand can be obtained [46,47]. In an arid or semi-arid area, the most important limiting factor is precipitation and species regeneration usually occurs during periods with abundant precipitation or sufficient water habitats [48]. These ecosystems are more vulnerable to human disturbances (grazing, land use/cover change, etc.) relative to other ecosystems [31,49]. Understanding what drives grazing impacts on population structure is critical for sustainability [50]. In this study, our results indicated that an increase in grazing pressure resulted in a decrease in the number of herbaceous and shrub species, shrub density, the number of *U. pumila* juvenile trees and seedlings, and grass biomass, which was not consistent with the study by Gaitán et al. [50], who found that grazing pressure negatively impacted the cover of palatable grasses and species richness but did not affect shrub cover. This indicates that grazing pressure significantly affected the stand structure of the *U. pumila*-dominated temperate savanna. Our results were similar to those of Li et al. [51], who also found that grass biomass and density of *U. pumila* seedlings significantly decreased with increased human disturbance.

A comparison of *U. pumila* density under different grazing pressures showed that many juveniles and seedlings were present under lightly-grazed conditions (Plots 1 and 2), and the size distributions of *U. pumila* under this condition had a reversed J shape (Figure 3). This result suggests the presence of favorable conditions for the establishment and survival of seedlings and for the continuous regeneration of the population. However, *U. pumila* populations under heavily grazed conditions (Plots 5 and 6) had a unimodal DBH distribution (Figure 3); very few juveniles were identified, which might not be offset by the longer lifespan of the adult trees. At each grazing pressure (Plots 1–6), adult *U. pumila* trees showed no obvious difference in density, suggesting that these trees had very strong anti-disturbance capability to grazing. Under moderately-grazed conditions (Plots 3 and 4), *U. pumila* could regenerate to a certain extent but the population density may decrease in the future. The continuation of heavy-grazing may lead to the disappearance of the *U. pumila*-dominated temperate savanna ecosystem Thus, over-utilization (for example, over-grazing and mowing) of the *U. pumila*-dominated temperate savanna may result in a decline of the *U. pumila* population in the Otindag sand land.

### 4.2. Ulmus Pumila Spatial Pattern and Influence of Grazing

Information about the regeneration of new individuals, the mortality of adults, and the overall demographics of a population following natural or human disturbance can be gained from investigating the spatial patterns of a species. This information can aid in understanding the ecological phenomena hidden in the spatial patterns [7,52]. Aggregation at a certain scale is a common pattern of species distribution in nature, which can result in the species resisting severe environments due to cluster effects; this can enable a stable population to continue to reproduce [53]. In our study, seedlings had a more aggregated distribution than juveniles or adult trees, occurring at scales <8–17 m for seedlings (Plots 1–4), <2–3 m for juvenile trees (Plots 1–4), and <1–2 m for the adult trees (Plot 1–6). This result suggests a random distribution for the adult *U. pumila* trees. Juvenile *U. pumila* trees had aggregation at the smaller scales and random distributions at the larger scales.

Interestingly, canopy diameter (Table 1) was about 7–8 m for the adult trees and 3–4 m for the juvenile trees, respectively, indicating that *U. pumila* seedlings may be successfully germinating under the canopies of adult trees. In arid and semi-arid areas, competition among seedlings for moisture and nutrients is relatively weak, a finding that has been frequently observed and is a response to patchy rainfall events in semi-arid savannas [25]. The aggregation pattern of *U. pumila* seedlings reflects their resistance to wind erosion and adaptation to the wind-sand environment, which may facilitate their growth and reduce the effects of sandstorms, thus improving their survival [19,54,55]. With the growth of *U. pumila* trees, increasing competition for water and soil nutrients led to self-thinning of the population. Adult and juvenile trees therefore had a random pattern at large scales. However, the aggregations for the adult and juvenile trees were evident at the small scales, which may be related to the initial distribution of the seedlings and sprouting reproduction due to human destruction.

In the lightly-grazed plots where seedlings were mostly unaffected by grazing, the aggregation distribution of seedlings may have been the result of seed dispersal or environmental heterogeneity [26,56,57]. The general characteristics of *U. pumila* seed shape and size (small and samara-shaped) reduce falling speed and enhance their dispersal capacity by wind. However, dispersal is often spatially limited, which leads to intra-specific aggregation [58]. In our study area, the prevailing wind-direction was from the northwest in the flowering season, which caused a spatial aggregation of seedlings to the southeast of the adult or juvenile trees (Figure 2). Directed dispersal can therefore generate apparent facilitative patterns of seedlings [54]. The aggregation may also have been the result of environmental heterogeneity, which causes uneven distribution of environmental variables both spatially and/or temporally [54]. In our study, a “fertility island” could have been created around the *U. pumila* trees due to plant litter and animal manure [35,59], which favored the growth of seedlings [60,61]. In addition, other investigations have found that micro-topography also affects the spatial patterns of species [62,63]. However, our plots were established in flat areas with a relatively low fluctuation (<1 m) to eliminate the effects of micro-topography. Therefore, the effects of micro-topography on the patterns of seedlings can be ignored.

Seasonal grazing and mowing in the moderately-grazed plots may lead to seedlings and juveniles being destroyed by the nonselective mowing activity in the autumn or a decline in density due to livestock feeding, thus further reducing spatial aggregation. This hypothesis may explain why numerous seedlings (predominantly germinated in the current year) were present in aggregations in August. Human disturbance therefore interrupts the chain of normal regeneration of *U. pumila* trees, which may eventually result in the breakdown of the *U. pumila* population in a semi-arid savanna ecosystem.

Overgrazing of livestock in heavily-grazed plots resulted in an extremely low density of seedlings and over-trampling from livestock affected tree regeneration. Due to this, seedling patterns in the heavily-grazed plots were not analyzed in this study. A comparison of patterns of *U. pumila* trees under different grazing pressures showed that there were no clear effects on the patterns for the adult trees. However, the effects were obvious for juveniles and seedlings. This implies that most juveniles are produced through coppice (sprouting from old stock) and can withstand trampling.

### 4.3. Spatial Associations among Woody Species

The spatial association of seedlings with juveniles was mainly positive at distances up to 4 m and the neutral at other distances. This finding suggests that seedlings were more likely to survive if their distance from other juveniles was less than 4 m. However, no significant spatial association between adults and seedlings at any scale were identified, presumably including water competition or grazing pressure. The areas under tree crowns are favored areas for animals to rest as they provide shelter from the summer heat and intense ultraviolet radiation. The soil under the canopy may also be destroyed due to trampling and recumbency, and at times a “bare soil circle” may be formed under trees [64]. All of this can lead to the hindrance of seedling survival. At the same time, adults had a significant positive association with juveniles on smaller scales, but not at other scales. We assume therefore that the associations between adults and juveniles may be caused by sprouting reproduction due to human destruction or originating from the associations between juveniles and seedlings.

The results for tree and shrub associations showed that there was almost a neutral association between the *U. pumila* adults and juveniles, and shrub species (including *C. microphylla* and *S. aquilegifolia*) (Figure 7). This finding suggests that the distance separating shrubs and trees had no significant effect on where they grew. Seedlings of *U. pumila* were negative or neutrally associated with *C. microphylla,* and positive associated with *S. aquilegifolia* (Figure 7). This finding may relate to water competition between the shrubs and seedlings. The association between *U. pumila* seedlings and *C. microphylla* shrubs suggests that *C. microphylla* may have adverse effects on the survival of *U. pumila* seedlings in areas with a high density of *C. microphylla*, i.e., competition correlation exists. However, in areas where the density of *C. microphylla* was lower, a significant correlation between *C. microphylla* and *U. pumila* seedlings did not exist, suggesting that there was no obvious competition for soil resources. The positive association between *U. pumila* seedlings and *S. aquilegifolia* shows that *S. aquilegifolia* may facilitate the regeneration of the *U. pumila* population.

### 4.4. Influence of Grazing on Shrub Encroachment

Although shrubs are often the dominant species in arid and semi-arid areas, their encroachment into rangelands has been considered an important indicator of degradation [65,66,67], further resulting in a decrease in grass productivity and biodiversity [68]. Although it is generally accepted that overgrazing is one of the main causes of shrub encroachment [69,70], recent findings have questioned the importance of overgrazing on shrub encroachment. Many studies have found that the reduction of grazing pressure after long-term grazing may be an important cause of shrub encroachment [71,72,73,74,75], which were consistent with our study findings. In the Otindag sand land, *C. microphylla* and *S. aquilegifolia* were the main species of shrub and additional similar results were also found: shrub density increased with decreasing grazing pressure (Table 1), with shrubs predominantly occurring in lightly-grazed plots (Plots 1 and 2). However, no shrubs were found in the heavily-grazed plots (Plots 5 and 6), which may be due to the abnormal germination and the regeneration of shrubs caused by over-trampling from livestock. Overgrazing in the heavily-grazed plots may reduce the productivity of the rangeland and result in edible shrubs being consumed, thus inhibiting their subsequent growth. After removing or reducing grazing pressures, through seasonal enclosure or other protective measures, bare land formed by grazing may be initially colonized by shrubs. This may be one reason why shrub density was higher in the lightly-grazed plots than in the other plots. Additionally, mowing in the autumn can remove shrub seedlings in the moderately-grazed plots, thus preventing shrub encroachment. Results from our investigation suggest that shrubs occurring in the lightly-grazed plots may be a result of encroachment due to decreased grazing pressure, similar to findings from previous studies. This finding also supported the suggestion that a shift in grazing pressure after long-term overgrazing is a direct factor that triggers shrub encroachment [69,76].

The mechanism of shrub encroachment to a new environment is very complex, which is closely related to the properties and functions of plants and their environment (such as precipitation pattern change, climate warming, grazing, etc.) [77]. The domestic herbivores by grazing can promote the dispersal of woody plant seeds, and the selective feeding by livestock reduced the grass biomass/cover, which led to reduction in competitiveness of herbaceous plants, and increase in competitiveness of woody plants [78]. In our study, the seeds of *C. microphylla* and *S. aquilegifolia* were dispersed by the feeding-excretion way or sticking to fur. Under the overgrazing, the livestock have to eat invasive shrub seedlings with better palatability because of fewer herbaceous plants, which makes it impossible to colonize and establish their communities. However, due to stronger competitiveness in shrubs than herbaceous plants, the reduced grazing pressure makes the shrub seedlings easier to survive and establish the stable communities, which enhance the shrub encroachments. Additionally, drought and precipitation variability are also the important factors that lead to shrub encroachments by enhancing the ability of competition and settlement of shrubs [79]. In this study area, drought is increasing continuously since 1960, especially during 1999–2011 when drought was the most serious period historically. Due to its deeper roots and stronger drought-tolerant than herbaceous plants, shrubs can utilize the deeper soil water during the drought, which promotes the growth and distribution of invaded shrubs. Under the combined effect of grazing and climate, shrubs expand continuously their distributions through self-reproduction and competition with the herbaceous species [79].

## 5. Conclusions

Because vegetation in fragile habitats is sensitive to environmental change and prone to degradation, *U. pumila*-dominated savanna in the Otindag sand land has been seriously degraded due to climate change. In addition, an increase in rangeland grazing pressures in recent decades has led to serious land degradation, and even to desertification of areas in this region [51]. In the heavily-grazed rangeland, the *U. pumila* population has declined and grasslands have become degraded. In the moderately-grazed rangeland, the *U. pumila* population was relatively stable but it suffered from poor seedling regeneration. In the lightly-grazed rangeland, the *U. pumila* population was increasing and it had good seedling regeneration, however serious shrub encroachment was occurring. The combination of our results indicates that controlled grazing is urgently needed in the Otindag sand land area to promote ecological restoration of the degraded *U. pumila*-dominated savanna ecosystem. Our findings show that light grazing is the optimum method for rangeland use and protecting the savanna ecosystem from further degradation. However, a shift in grazing pressures to a light-grazing situation after long-term overgrazing may reduce the availability of rangeland; therefore, we propose that a moderate-grazing regime is required. Reformation of property rights of the commons (heavily-grazed rangeland) is needed to reduce rangeland pressures and to avoid further degradation [80,81]. This investigation provides important information for management practices and planning, and specific recommendations for the *U. pumila*-dominated savanna. It is important to avoid planting woody shrub species that have negative interspecific associations (*C. microphylla* vs. *U. pumila seedling*) in homogeneous habitats. The presence of *S. aquilegifolia* and *U. pumila* seedlings together shows that *S. aquilegifolia* may be a more suitable shrub vegetation type to promote savanna restoration in this area.

## Figures and Tables

**Table 1:** Vegetation information for Plots 1–6.

Grazing Pressure	Plot	No. Herbaceous Species	Aboveground Herbaceous Dry Biomass/g·m^−2^	Woody Species	Category	Number	Average DBH/cm	Average Height/m	Average Crown Diameter/m
Light	1	20	127.63 ± 20.22	*U. pumila* trees	Adult trees	75	26.42 ± 4.95	7.54 ± 1.79	6.69 ± 1.41
					Juvenile trees	139	15.44 ± 4.61	3.78 ± 1.21	3.64 ± 1.09
					Seedlings	106	/	0.87 ± 0.27	1.18 ± 0.43
				Shrubs	*C. microphylla*	157	/	0.44 ± 0.17	0.63 ± 0.21
					*S. aquilegifolia*	114	/	0.70 ± 0.24	0.98 ± 0.36
					*S. linearitipularis*	2	/	1.82 ± 1.60	1.94 ± 1.85
	2	21	225.85 ± 13.65	*U. pumila* trees	Adult trees	83	24.25 ± 4.47	7.04 ± 1.38	6.17 ± 1.05
					Juvenile trees	104	14.34 ± 3.32	3.96 ± 0.54	3.78 ± 0.76
					Seedlings	101	/	0.81 ± 0.27	1.17 ± 0.55
				Shrubs	*C. microphylla*	55	/	0.55 ± 0.14	0.68 ± 0.19
					*S. aquilegifolia*	109	/	0.50 ± 0.16	0.56 ± 0.21
					*S. linearitipularis*	25	/	1.80 ± 0.64	2.06 ± 0.75
Moderate	3	15	112.95 ± 5.09	*U. pumila* trees	Adult trees	86	26.81 ± 5.11	7.70 ± 0.97	7.12 ± 1.81
					Juvenile trees	73	13.98 ± 6.45	3.48 ± 1.83	3.23 ± 1.74
					Seedlings	89	/	0.75 ± 0.28	0.92 ± 0.39
				Shrubs	*C. microphylla*	24	/	0.79 ± 0.22	0.76 ± 0.21
					*S. aquilegifolia*	19	/	0.79 ± 0.29	0.80 ± 0.30
	4	19	218.23 ± 12.81	*U. pumila* trees	Adult trees	78	28.24 ± 5.31	7.42 ± 0.87	7.61 ± 1.46
					Juvenile trees	55	16.60 ± 5.85	3.22 ± 1.20	2.75 ± 1.29
					Seedlings	67	/	0.71 ± 0.36	0.90 ± 0.35
				Shrubs	*C. microphylla*	14	/	0.34 ± 0.10	0.70 ± 0.40
Heavy	5	14	80.84 ± 4.33	*U. pumila* trees	Adult trees	82	28.18 ± 6.18	7.72 ± 1.47	7.11 ± 2.14
					Juvenile trees	18	18.37 ± 3.72	4.37 ± 1.32	3.91 ± 0.89
					Seedlings	6	/	0.92 ± 0.72	1.07 ± 0.91
	6	15	103.02 ± 9.65	*U. pumila* trees	Adult trees	84	28.57 ± 4.94	7.95 ± 1.35	7.18 ± 1.88
					Juvenile trees	11	18.25 ± 1.23	4.31 ± 1.39	4.06 ± 1.23
					Seedlings	9	/	0.94 ± 0.84	1.01 ± 0.87
